# Symbiotic fungi from a wild grass (*Celtica gigantea*) increase the growth, grain yield and quality of tritordeum under field conditions

**DOI:** 10.1093/aobpla/plae013

**Published:** 2024-03-15

**Authors:** Iñigo Zabalgogeazcoa, Juan B Arellano, Elena Mellado-Ortega, Francisco Barro, Ana Martínez-Castilla, Virginia González-Blanco, Beatriz R Vázquez de Aldana

**Affiliations:** Unit of Plant-Microorganism Interactions, Institute of Natural Resources and Agrobiology of Salamanca, Spanish National Research Council (IRNASA-CSIC), Cordel de Merinas 40-52, 37008 Salamanca, Spain; Unit of Plant-Microorganism Interactions, Institute of Natural Resources and Agrobiology of Salamanca, Spanish National Research Council (IRNASA-CSIC), Cordel de Merinas 40-52, 37008 Salamanca, Spain; Unit of Plant-Microorganism Interactions, Institute of Natural Resources and Agrobiology of Salamanca, Spanish National Research Council (IRNASA-CSIC), Cordel de Merinas 40-52, 37008 Salamanca, Spain; Department of Biology, Duke University, 130 Science Dr, Durham, NC 27710, USA; Department of Plant Biotechnology, Institute for Sustainable Agriculture, Spanish National Research Council (IAS-CSIC), Avenida Menéndez Pidal s/n, Campus Alameda del Obispo, 14004 Córdoba, Spain; Department of Plant Biotechnology, Institute for Sustainable Agriculture, Spanish National Research Council (IAS-CSIC), Avenida Menéndez Pidal s/n, Campus Alameda del Obispo, 14004 Córdoba, Spain; Unit of Plant-Microorganism Interactions, Institute of Natural Resources and Agrobiology of Salamanca, Spanish National Research Council (IRNASA-CSIC), Cordel de Merinas 40-52, 37008 Salamanca, Spain; Unit of Plant-Microorganism Interactions, Institute of Natural Resources and Agrobiology of Salamanca, Spanish National Research Council (IRNASA-CSIC), Cordel de Merinas 40-52, 37008 Salamanca, Spain

**Keywords:** Endophyte, field trial, fungal inoculation, grain yield, grain quality, symbiosis, tritordeum

## Abstract

Abstract. Plants function in symbiosis with numerous microorganisms, which might contribute to their adaptation and performance. In this study, we tested whether fungal strains in symbiotic interaction with roots of *Celtica gigantea*, a wild grass adapted to nutrient-poor soils in semiarid habitats, could improve the field performance of the agricultural cereal tritordeum (*Triticum durum* × *Hordeum chilense*). Seedlings of tritordeum were inoculated with 12 different fungal strains isolated from roots of *Celtica gigantea* that were first proved to promote the growth of tritordeum plants under greenhouse conditions. The inoculated seedlings were transplanted to field plots at two locations belonging to different climatic zones in terms of mean temperatures and precipitation in the Iberian Peninsula. Only one strain, *Diaporthe iberica* T6, had a significant effect on plant height, number of tillers and grain yield in one location. This result showed a substantial divergence between the results of greenhouse and field tests. In terms of grain nutritional quality, several parameters were differentially affected at both locations: *Diaporthe* T6, *Pleosporales* T7, *Zygomycota* T29 and *Zygomycota* T80 increased the content of total carotenoids, mainly lutein, in the colder location; whereas gluten proteins increased with several treatments in the warmer location. In conclusion, early inoculation of tritordeum plants with fungal symbionts had substantial beneficial effects on subsequent plant growth and development in the field. Regarding grain nutritional quality, the effect of inoculation was affected by the agroclimatic differences between both field locations.

## Teaser

We studied in two locations if fungal strains isolated from roots of a wild grass adapted to nutrient-poor soils could improve the field performance of tritordeum. Of the 12 strains assayed, only *Diaporthe iberica* increased plant growth and grain yield in one location. The other strains, which were selected for their ability to promote plant growth under greenhouse conditions, did not improve tritordeum growth under field conditions. These results show a substantial divergence between greenhouse and field tests. Some effects of fungal inoculation on grain nutritional quality were observed and depended on the location.

## Introduction

Plants function in association with a complex set of symbiotic microorganisms, which constitute their microbiome. In turn, the microbiome can perform functions that contribute to plant adaptation and performance (Vandenkoornhuyse *et al*. 2015). Mechanization, plant breeding and agrochemistry produced huge technical developments in agriculture in the 20th century, but the contribution of the microbiome to plant performance was largely overlooked as a technological front. This occurred because the current notion of plants as holobionts was largely ignored in the 20th century (Suárez 2018). Under this new perspective, microorganisms that fulfill functions related to plant nutrition and defense represent potential tools for increasing plant productivity, and an alternative to the excessive reliance on agrochemicals (Rodriguez *et al.* 2008; Pozo *et al*. 2021; Trivedi *et al.* 2021). Therefore, unveiling particular functions of microbiome components in plant performance opens new possibilities for harnessing microorganisms for technological advances in agriculture (Pozo *et al.* 2021; Trivedi *et al.* 2021; Ali *et al.* 2022).

A large body of published research has shown that plant microbiome components improve plant nutrition, yield, and tolerance to abiotic and biotic stresses (Pereira *et al*. 2021; Ali *et al.* 2022; Chaudhary *et al.* 2022; Meena *et al.* 2023). However, most of these studies consisted of short-term greenhouse or laboratory assays where individual or few (consortia) microbial species were inoculated into plants, often in pre-sterilized soils. The actual agricultural effectivity of microbes identified in such studies could be questioned because microbiomes are very complex sets of interacting microorganisms that respond to the environment as well as to the plant genotype (Li *et al.* 2021; Escudero and Bulgarelli 2023). As a result, the location, time of the year or plant species can determine the structure of a microbiome in terms of the diversity and abundance of its components (Bastías *et al.* 2022). In addition, microbe-to-microbe interactions also affect microbiome structure and composition. For example, ‘keystone’ microbial species can positively or negatively affect the abundance of other microbial species (Markalanda *et al.* 2022). Therefore, in contrast to experiments conducted under greenhouse-controlled conditions, plants in field experiments are exposed to a much more variable environment, and to a rich soil and aerial microbiota. Such conditions might impose significant constrictions on the success of inocula selected under controlled conditions. For this reason, field testing holds great importance in evaluating the performance of microbial strains for plant growth promotion under real-world agricultural conditions. In this work, we delved into the field performance of a set of fungal strains capable of promoting plant growth in short-term greenhouse experiments

Tritordeum is a hybrid cereal developed from a cross between durum wheat (*Triticum turgidum*) and *Hordeum chilense* (Martín *et al.* 1999). This cereal has some unique nutritional properties like a high content of oleic acid, up to six times more lutein than common wheat, a content of gluten proteins 50% lower than wheat, and 30% more dietary fiber than wheat (Atienza *et al.* 2007; Vaquero *et al.* 2018). Wheat-based foods provide around 40% of dietary fibre and have a significant contribution to the intake of vitamins, micronutrients and proteins in consumers (Shewry and Hyde 2015). Therefore, changes in yield or grain quality can affect the dietary health of millions of people (Galani *et al.* 2022).

In a previous study, several fungal strains isolated from surface̶ disinfected roots of the wild grass *Celtica gigantea* Link (=*Stipa gigantea*) promoted the growth of tritordeum plants in a short-term greenhouse assay (Vázquez de Aldana *et al.* 2021). *Celtica gigantea* is a large perennial grass native to the Iberian Peninsula that grows in semiarid habitats characterized by rocky sandy soils where nutrient and water availability is scarce (Vázquez and Devesa 1996). The objective of this study was to find out whether fungal strains isolated from *C. gigantea* would affect plant development, grain production and nutritional grain quality of tritordeum under agricultural field conditions. For this purpose, fungi associated with *C. gigantea* roots were selected from a culture collection, and field experiments were carried out in two locations with different agroclimatic characteristics.

## Materials and Methods

### Fungal strains and production of fungal inoculum

Eleven fungal strains isolated from surface-disinfected roots of *C. gigantea* were selected for field testing (Vázquez de Aldana *et al.* 2021) (Supporting information Table S1). The following strains were selected because they conferred beneficial effects on tritordeum plants in a short-term greenhouse bioassay: *Diaporthe* T6, *Zygomycota T80*, *Basidiomycota* T40, *Dothideomycota T10*, *Microphaeropsis* T33, *Alternaria A60,* and *Unknown* T18 caused growth promotion; while *Collembolispora* T17, *Zygomycota T29*, *Diaporthe* T61 and *Pleosporales T7* increased leaf nutrient content (Vázquez de Aldana *et al.* 2021). In addition, *Diaporthe* FR9, isolated from roots of *Festuca rubra*, and having a favourable effect on tritordeum growth in the greenhouse (unpublished data), was also assessed for plant growth promotion.

To produce fungal inoculum, 30 g of sugar beet pulp pellet were mixed with 9.0 g CaCO_3_, 4.5 g CaSO_4_ and 60 mL of water, and autoclaved in wide-mouth glass bottles (Vázquez de Aldana *et al.* 2020). Each bottle of beet pulp medium was inoculated with a fungal strain, and cultured at room temperature (20–22 °C) for 4 weeks. After this time, one part of each fungal inoculum was mixed with seven parts (v/v) of a 1:1 (v/v) mixture of peat moss (Gramflor, Valencia, Spain) and perlite (Gramflor, Valencia, Spain) previously treated at 80 °C for 24 h. The substrates containing fungal inoculum were used to germinate and inoculate tritordeum seedlings.

### Field experiments

**Figure 1.**
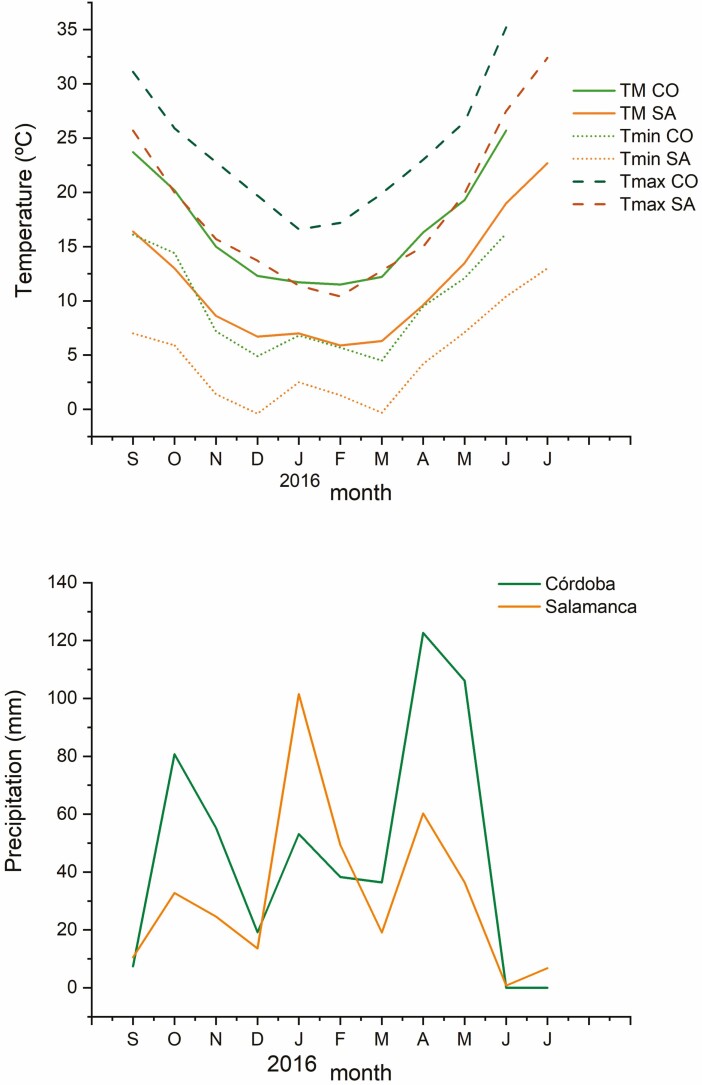
Climatic characteristics of the field plot locations during the 2015–16 period. *T*_M_ = mean temperature; *T*_min_ = mean minimum temperature; *T*_max_ = mean maximum temperature.

To evaluate the effect of fungal strains on the field performance of tritordeum, two field experiments were performed simultaneously at two locations 350 km apart in Spain: Salamanca (40°54’16’’N; 5°46’32’’W) and Cordoba (37°51’54’’N; 4°47’53’’W). These locations belong to two different climatic zones according to Köppen-Geiger classification for the Iberian Peninsula (AEMET, 2011). Córdoba is about 6°C warmer than Salamanca during all the year (Fig. 1). The average annual precipitation during the period 1970-2000 was lower in Salamanca (381 mm) than in Córdoba (543 mm) (AEMET, 2011), and this difference is especially marked in autumn and spring (Fig. 1). From now on we will refer to Córdoba as the warmer location and to Salamanca as the colder location.

A completely randomized design with six replicates was used. Each replication contained 13 plots, 12 for each fungal treatment plus an uninoculated control treatment. Each plot consisted of a line with 10 plants spaced by 10 cm, and a space of 40 cm was left between plots. To inoculate the plants, 2–3 seeds of tritordeum cv. Aucan were sown in 20 mL coconut fiberpots (Cocopot, Valencia, Spain) filled with the substrate containing the inoculum above described. The control plants were germinated in a substrate without inoculum. After germination, only one plant was left on each fiberpot. Two weeks after germination, individual potted plants were transplanted to their respective plots. The field transplants were made on 9 November 2015, in Salamanca, and on 12 November 2015, in Córdoba. Both experiments were conducted under rainfed conditions. At each experimental location, a soil sample was collected at transplantation time for chemical analysis by official standard methods at IRNASA-CSIC. Soil from Córdoba had greater organic matter and lower N content than the one from Salamanca (see Supporting Information—Table S1).

**Figure 2.**
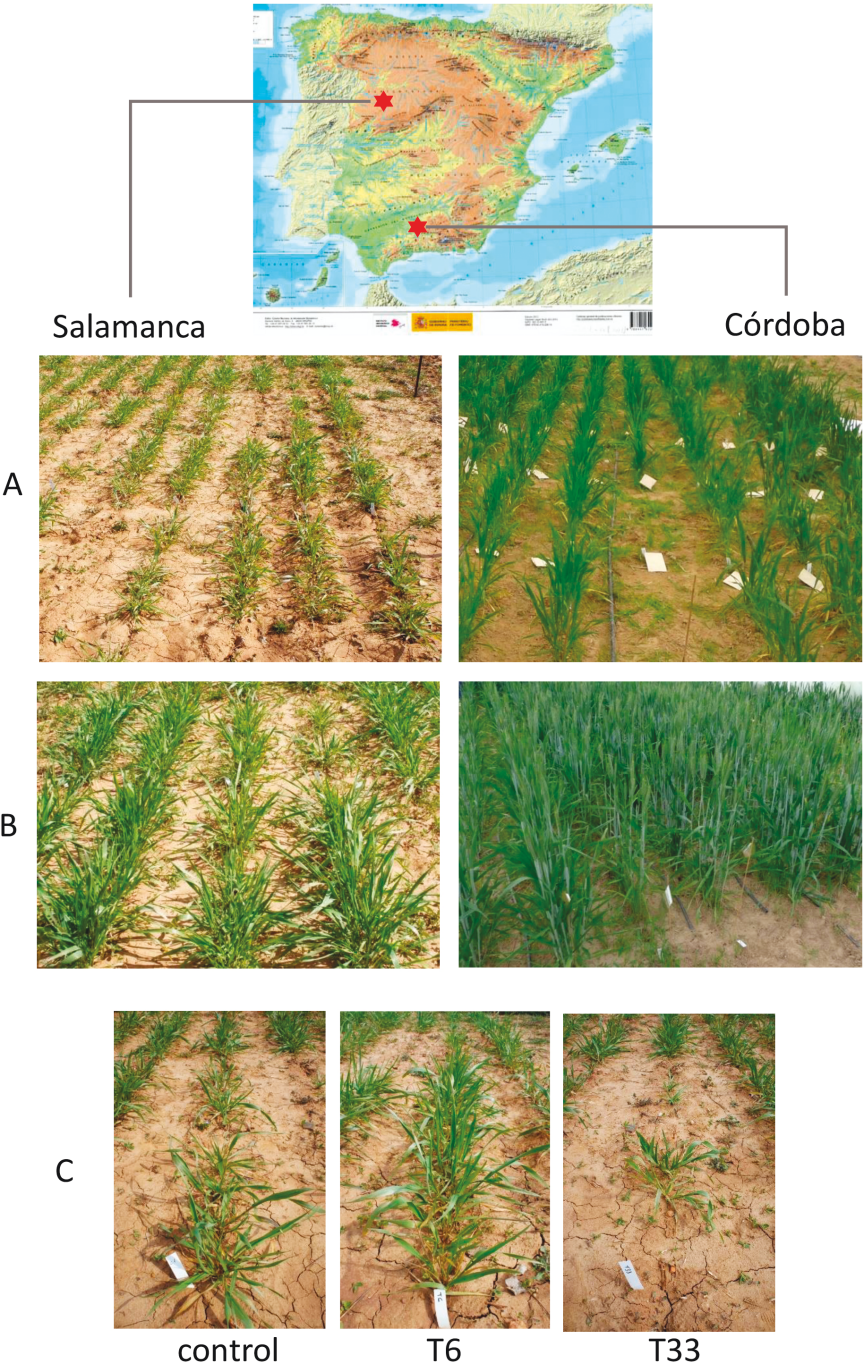
Details of tritrodeum plants in Salamanca, the colder location, (40°54ʹ16″ N, 5°46ʹ32 ″ W) and in Córdoba, the warmer location (37°51ʹ54″ N 4°47ʹ53″ W). (A) Plants at the time when height and tiller number were measured, March 13 in Salamanca and February 9 in Córdoba. (B) Tritordeum plants on April 8 showing the difference in the phenological stage of plants at both locations. (C) Appearance of plots with tritrodeum plants with different inoculation treatments: control (uninoculated), *Diaporthe* T6 and *Microphaeropsis* T33, in Salamanca. Each plot consisted of a line with 10 plants, each separated by 10 cm. (Origin of the map image: ‘Instituto Geografico Nacional’ of Spain).

Plant height, measured as the length from the base of the stem to the tip of the top leaf, and tiller number were recorded on 8 February 2016 in the warmer location (Córdoba) and on 13 March 2016 in the colder one (Salamanca), when plants were at a growth stage equivalent to stage 5 on the Feekes scale for wheat (Fig. 2). When plants had five leaves fully expanded, a sample was collected for nutrient analysis. For this purpose, the third leaf was collected from a tiller of each plant, the leaves of the ten plants of each plot were pooled in a single sample and freeze-dried and ground for chemical analyses. Plants were harvested at maturity and the number of heads per plant, grain weight, and 1000 grain weight were determined. A grain subsample was ground for chemical analyses.

### Chemical analyses

Leaf samples were analysed for mineral elements and total antioxidant capacity, and grain samples were for nutritional quality parameters. For mineral elements analysis, samples were calcined at 450 °C for 8 h and the ashes dissolved in HCl:HNO_3_:H_2_O (1:1:8). The concentrations of P, K, Ca, Mg, S, Mn, Fe, Cu and Zn were determined by inductively coupled plasma atomic emission spectroscopy (ICP-OES, Varian 720-ES). Protein was determined as nitrogen using a C/H/N analyser (LECO CN628). The fibre content was determined by analysing neutral detergent fibre (NDF), acid detergent fibre (ADF) and lignin using the filter bag technique with an automated fibre analyser (Ankom A2000). Trolox equivalent antioxidant capacity (TEAC) was determined in lyophilized plant material using the oxygen radical absorbance capacity (ORAC) method described (Gillespie *et al.* 2007) with some modifications (Mellado-Ortega *et al.* 2017). Total phenolic content was determined spectrophotometrically according to the Folin-Ciocalteau method (Ainsworth and Gillespie 2007), using gallic acid as the reference standard. Carotenoid pigments were extracted using acetone and subsequently, the pigment composition was analysed using high performance liquid chromatography (HPLC) equipment with a UV–visible detector as described (Mellado-Ortega and Hornero-Méndez 2017). Grain protein fractions, gliadins and glutenins, were quantified by HPLC as described by Marín-Sanz *et al.* (2020). Soluble sugars were quantified by HPLC according to the standard Association of Official Analytical Chemists method 2000.17 (AOAC 2009). The starch content of whole flour was determined according to standard International Association for Cereal Chemistry method no. 123/1 (ICC 1994). Fatty acids were quantified by gas chromatography according to the standard COI/T.20/Doc. No 33 method (IOC 2017).

### Statistical analyses

For the statistical analysis, we used a data matrix of 13 treatments × 2 locations × 6 replications, that is, for each plot we considered the mean of 10 plants within the plot. The effect of inoculation treatment as a fixed factor on plant growth and grain yield parameters was evaluated by means of one-way ANOVA for each field location. Post hoc comparisons of means were made using the Holm–Sidak test. The Shapiro–Wilk normality test and the Brown–Forsythe equal variance test were used to determine if the data met ANOVA assumptions. When ANOVA assumptions were not met (number of heads and grain yield datasets), non-parametric Kruskal–Wallis and Dunn´s test for post hoc comparison of means were used. To determine relationships between the leaf nutrient content and plant growth and grain yield parameters, correlation analyses were performed across all inoculation treatments for each field location. To analyse the effect of inoculation treatment on plant growth and grain production parameters, a principal component analysis (PCA) was performed using matrix correlation including the following parameters from both locations: plant height and tiller number in March, number of heads per plant, grain production and weight of 1000 grains. To analyse the overall effect of the inoculation treatment on the grain nutritional quality a PCA was performed with a set of inoculation treatments that improved plant growth. This multivariate analysis included a total of 24 variables from a whole chemistry analysis of the grain samples: fat, starch, protein, fibers (ADF, NDF, lignin), dry matter digestibility, total soluble sugars, gliadins, glutenins, prolamins, total lutein, carotenoids, total phenolic compounds, TEAC and macronutrients (P, K, Ca, Mg and S) and micronutrients (Mn, Fe, Cu and Zn). All statistical analyses were performed with SigmaPlot 14.5 and IBM SPSS Statistic 27.

## Results

### Effect of fungal inoculation on plant growth and grain yield

**Figure 3.**
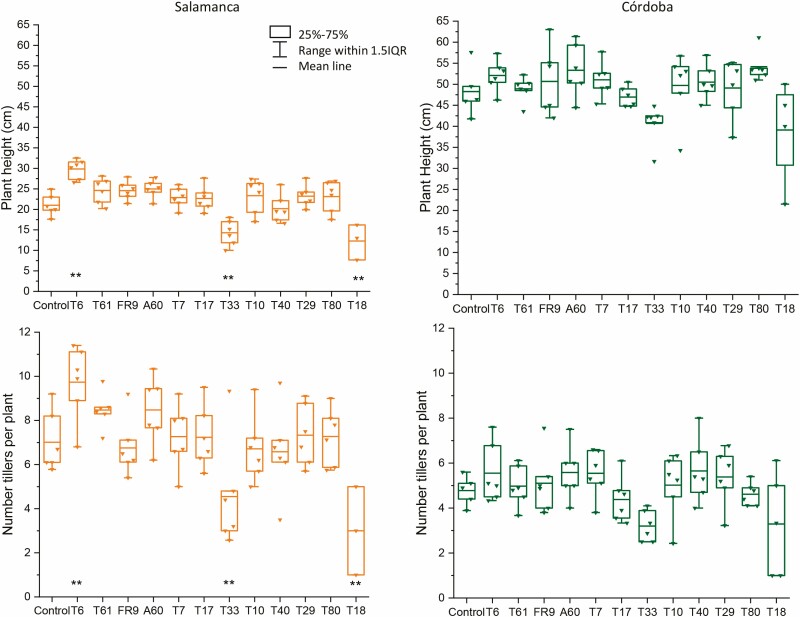
Plant height and number of tillers in March of tritordeum cv. Aucan plants inoculated with different fungal strains in the colder (Salamanca) and the warmer (Córdoba) locations. *Diaporthe* T6, T61, FR9; *Alternaria* A60; *Pleosporales* T7; *Collembolispora* T17; *Paraconiothirium* T33; *Dothideomycota* T10; *Basidiomycota* T40*; Zygomycota* T29, T80; *Unknown* T18, inoculated control. Statistically significant differences versus control are marked as **P < 0.01.

The phenotypic plasticity of tritordeum was evidenced in the different growth patterns observed between locations. The plants from the warmer location (Córdoba x̄= 48.98 ± 1.16 cm) were about two times (*P* < 0.001) as tall as those from the colder one (Salamanca x̄= 22.11 ± 1.16 cm) (Figs. 2 and 3). Contrary to this trend, tillering was significantly (*P* < 0.001) greater in the colder (x̄= 6.96 ± 0.46 tillers per plant) than in the warmer location (x̄= 4.81 ± 0.24 tillers per plant). Differences among inoculation treatments were statistically significant in both locations for plant height (Salamanca *F*_(12,74)_ = 10.659, *P* < 0.001; Córdoba *F*_(12,74)_ = 2.812, *P* = 0.004) and tiller number (Salamanca *F*_(12,74)_ = 5.579, *P* < 0.001; Córdoba *F*_(12,74)_ = 2.993, *P*  = 0.002). However, only *Diaporthe* T6 in the colder location produced plants significantly taller (x̄ = 29.81 ± 0.64 cm) and with a greater number of tillers (x̄ = 9.71 ± 0.46) than the control (x̄ = 21.02 ± 0.78 cm; x̄ = 7.03 ± 0.51 tillers) (Fig. 3). In the warmer location none of the inoculation treatments differed significantly (*P* > 0.05) from the control. Plant mortality was high at both locations for the *Paraconiothirium T33* and *Unknown* T18 treatments. Although these fungi gave satisfactory results in a greenhouse bioassay (Vázquez de Aldana *et al.* 2021), both were deleterious in the field experiments (Fig. 3).

**Figure 4.**
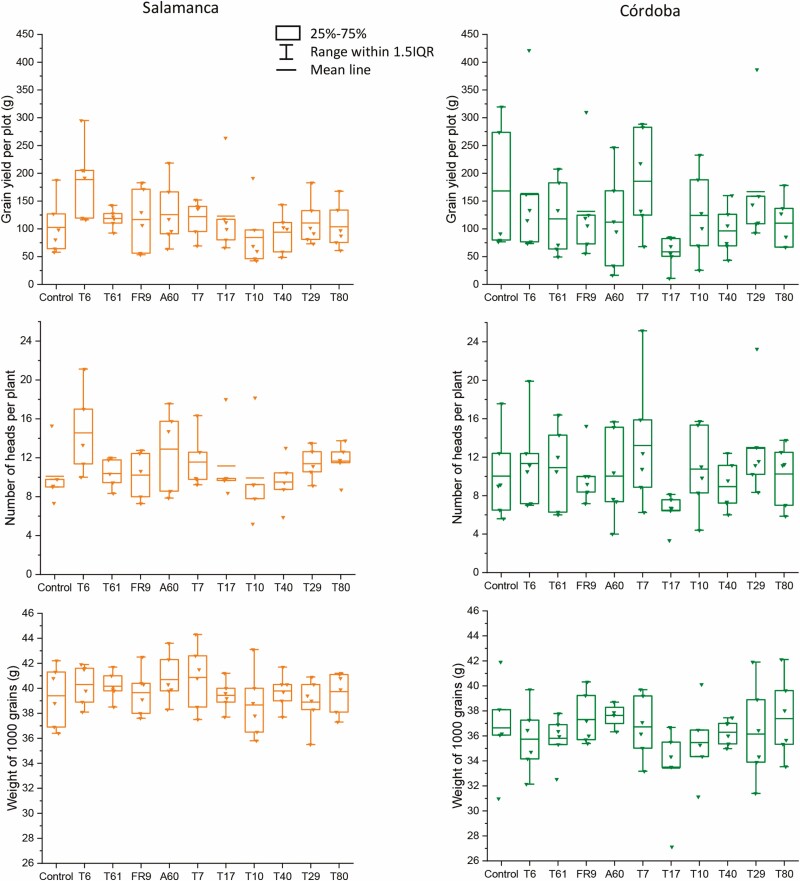
Number of heads per plant, weight of 1000 grains and grain production (as weight of grains per plot) of tritordeum cv. Aucan plants inoculated with different fungal strains, in the colder (Salamanca) and the warmer (Córdoba) locations. *Diaporthe* T6, T61, FR9; *Alternaria* A60; *Pleosporales* T7; *Collembolispora* T17; *Paraconiothirium* T33; *Dothideomycota* T10; *Basidiomycota* T40*; Zygomycota* T29, T80; *Unknown* T18, uninoculated control.

At harvest time, the number of heads per plant did not differ (*P* > 0.05) between the warmer location (Córdoba x̄= 10.44 ± 0.53 heads) and the colder one (Salamanca x̄= 10.88 ± 0.40 heads). Similarly, grain yield per plot did not significantly differ between the warmer (x̄= 129.92 ± 10.74 g) and the colder location (x̄= 117.27 ± 6.47 g). In the colder location the number of heads (*F*_1,11_ = 6.611, *P* = 0.028) and grain yield per plot (*F*_1,11_ = 6.603, *P* = 0.028) of the *Diaporthe* T6 treatment (x̄= 14.74 ± 0.98 heads; x̄= 22.05 ± 3.54 g) were significantly greater than those of the control (x̄= 9.25 ± 0.89 heads; x̄= 13.69 ± 2.82 g) (Fig. 4). This significance level was obtained when only the control and *Diaporthe* T6 treatments were included in the analysis. In the warmer location, no inoculation treatment was statistically significant for those parameters. The weight of 1000 seeds was significantly (*P* < 0.01) greater in Salamanca (x̄= 39.78 ± 2.65 g) than in Córdoba (x̄= 36.27 ± 2.65 g); however, it did not differ (*P* > 0.05) among treatments at each location (Fig. 4).

**Figure 5.**
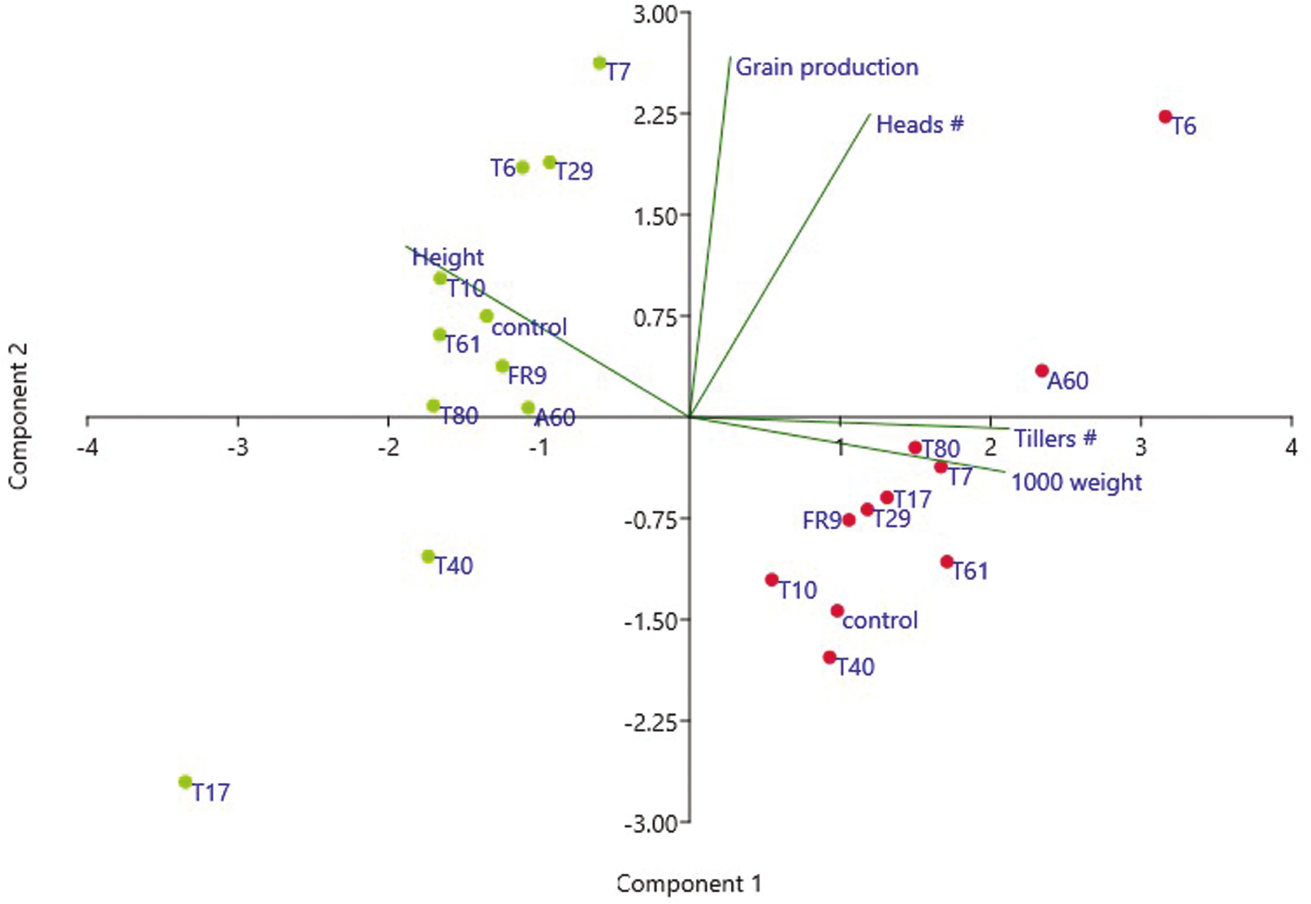
Principal component analysis of growth and grain yield parameters of tritordeum cv. Aucan plants inoculated with different fungal strains in the colder (Salamanca, red dots) and the warmer (Córdoba, green dots) locations. Each dot in the plot indicates the inoculation treatment: *Diaporthe* T6, T61, FR9; *Alternaria* A60; *Pleosporales* T7; *Collembolispora* T17; *Dothideomycota* T10; *Basidiomycota* T40*; Zygomycota* T29, T80; control = uninoculated. 1000 weight = weight of 1000 grains. A colour version of this figure appears in the online version of this article.

The overall effect of the inoculation treatments on all the plant growth and grain yield parameters at both field locations was analysed by means of a PCA. The *Paraconiothirium T33* and *Unknown* T18 treatments were not included in this analysis because both caused high plant mortality. The principal components (PC) 1 and 2 accounted for 56.5% and 37.0% of the total variance, respectively. The ordination of samples on the plane defined by PC1 and PC2 showed a clear segregation associated with traits derived from phenotypic plasticity along the PC1 (Fig. 5). The samples from the colder and drier location (Salamanca) were located in the positive part of the PC1, characterized by the tiller number in March and weight of 1000 grains. Samples from the warmer and more humid location (Córdoba) were clustered in the negative part of the PC1, which was mainly related to plant height in March (Fig. 5). In turn, samples were distributed along the PC2 according to the inoculation treatment, and they were ranked mainly according to their grain yield and number of heads. Thus, the treatments with the highest values in grain yield and number of heads were *Diaporthe* T6 in the colder location, and *Pleosporales T7*, *Diaporthe* T6 and *Zygomycota T29* in the warmer location (Fig. 5). The PCA results also showed that inoculation treatments were more effective in the warmer location, as indicated by the position of the control respect to the inoculation treatments.

### Effect of fungal inoculation on leaf mineral content

**Figure 6.**
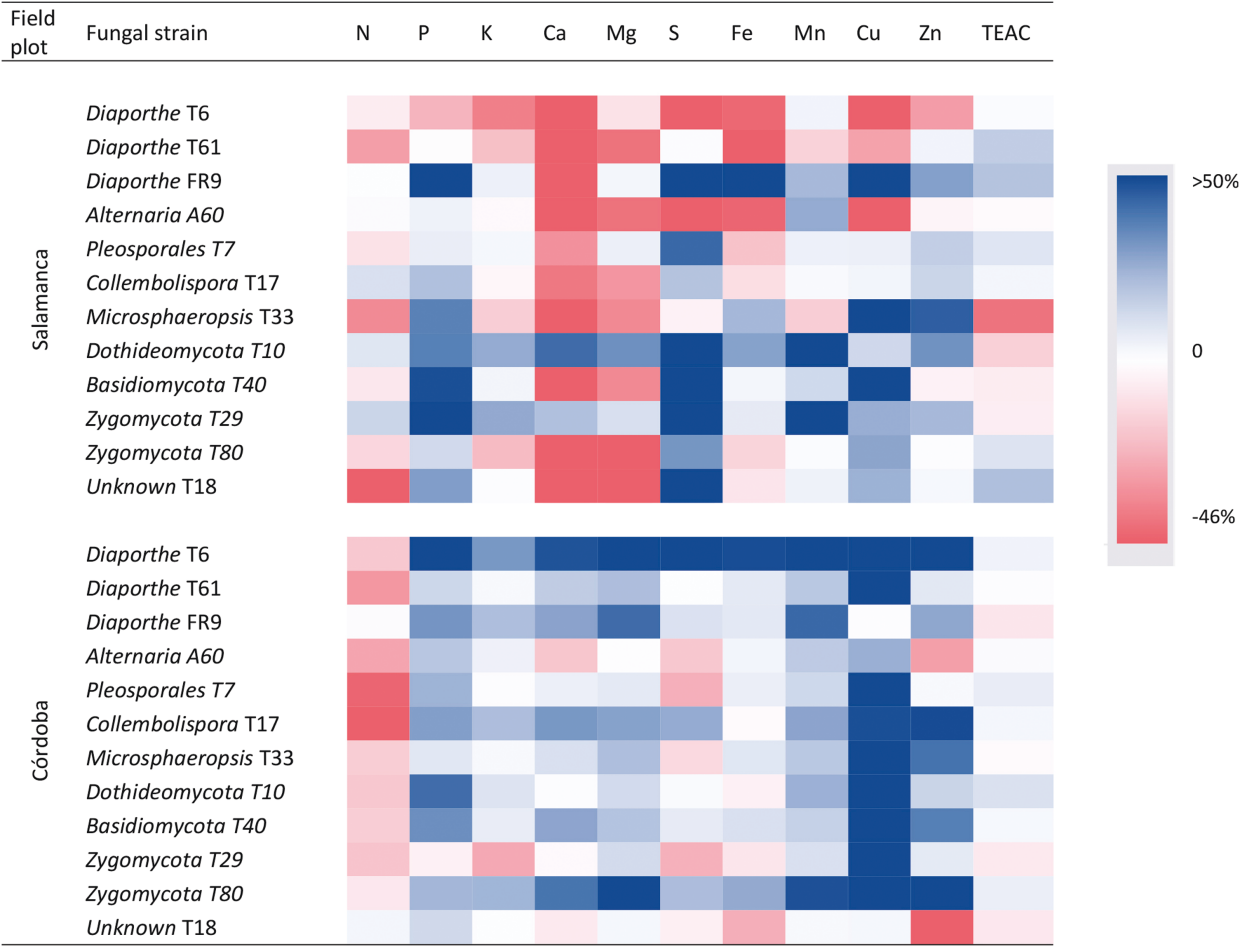
Effect of fungal inoculation in the nutrient content and Trolox equivalent antioxidant capacity (TEAC) of tritordeum leaves at the vegetative stage in the colder (Salamanca) and the warmer (Córdoba) locations. The colour is based on percent of variation respect to uninoculated controls.

The effect of fungal inoculation on the nutrient content of leaves varied between locations (Fig. 6). In the colder location, most inoculation treatments increased P, S and Cu contents; *Dothideomycota T10* increased all nutrient contents but N, and *Zygomycota T29* increased all nutrients except Mg and Fe. In the warmer, location most inoculation treatments increased P, Mn and Cu concentrations; *Diaporthe* T6 and *Zygomycota T80* treatments increased all nutrient contents but N (Fig. 6). In addition, *Diaporthe* FR9, *Diaporthe* T61 and *Unknown* T18 increased the total antioxidant capacity in Salamanca. Although leaf samples were collected at the same vegetative stage in both locations, there was a phenological gap between them due to differences in soil parameters and climatic conditions between locations (Fig. 1; see Supporting Information—Table S2). Plants grown in the warmer showed faster development than plants grown in the colder one (Fig. 2). Plants grown in Cordoba had significantly (*P* < 0.001) higher N, Ca, K, S and micronutrient (Fe, Mn, Cu and Zn) content, but lower P, Mg and TEAC than plants from Salamanca (**see** Supporting Information—Table S3).

### Effect of fungal inoculation on grain quality

**Figure 7.**
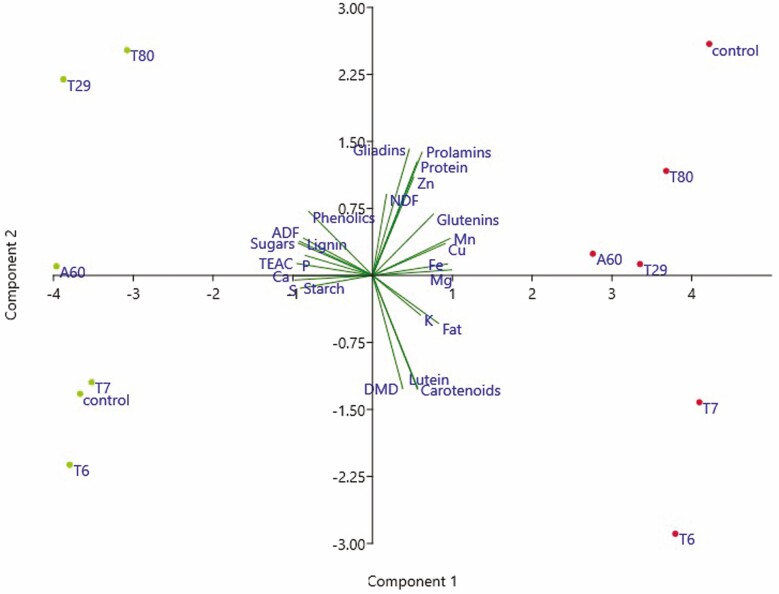
Principal component analysis of grain quality traits of tritordeum cv. Aucan plants in the colder (Salamanca, red dots) and the warmer (Córdoba, green dots) locations. Selected inoculation treatments: *Diaporthe* T6, *Pleosporales T7, Alternaria* A60, *Zygomycota* T29 and T80, control = uninoculated. Dispersion of samples in the plane defined by principal components 1 and 2. TEAC = Trolox equivalent antioxidant capacity; DMD = dry matter digestibility; ADF = acid detergent fibre; NDF = neutral detergent fibre. A colour version of this figure appears in the online version of this article.

Grain samples from the most effective growth treatments (*Diaporthe* T6, *Pleosporales T7*, *Alternaria A60*, *Zygomycota T29* and *Zygomycota T80*) were analysed for several nutritional quality parameters (**see** Supporting Information—Table S4 and S5). The effect of fungal inoculation on grain quality was assessed by means of a PCA (Fig. 7; **see** Supporting Information—Fig. S1). The ordination of samples on the factorial plane delimited by PC1 and PC2 accounted for 61.3% and 14.2% of the total variance, respectively. Two clusters based on the field location were clearly distinguished (Fig. 7). The samples from the warmer location (Córdoba) were mainly characterized by soluble sugars, starch, ADF, total phenolic compounds, TEAC, P, Ca, S and lignin. In contrast, samples from the colder location (Salamanca), clustered in the positive part of the PC1, were associated with fat, glutenins, K, Mg and micronutrients (Fe, Mn and Cu). In turn, samples were distributed along the PC2 according to the inoculation treatment. Treatments with *Diaporthe* T6 and *Pleosporales T7* were in the negative part of the PC2, characterized by total lutein, total carotenoids and DMD (Fig. 7). The *Zygomycota T80*, *Zygomycota T29* and *Alternaria A60* treatments were in the positive part of the PC2, characterized by active gliadins, total prolamins, protein, Zn and NDF. Thus, at both field locations the ranking according to the inoculation treatment was similar, except for non-inoculated controls that were in opposite quadrants.

In response to inoculation treatments, the gluten proteins, direct indicators of the technological properties of cereals, exhibited varying degrees of alteration depending on the location. While in the colder location, all the gliadin fractions showed values similar to or below the control, in the warmer one, these values were higher than the control. The same occurred with glutenin fractions and total prolamins, which in all cases showed higher values than the untreated control in the warmer location. As the different plant height and tillering phenotypes were observed at each location, these characteristics could be an expression of plasticity. Regarding fatty acid profiles, most of these compounds were not affected by the inoculation treatment (**see** Supporting Information—Table S5). Only the *Alternaria A60* treatment stood out due to an increase in mystiric, palmitic, trans–oleic and trans–linoleic fatty acids in the colder location.

### Effect of fungal inoculation on plant growth, grain yield and nutritional quality

**Figure 8.**
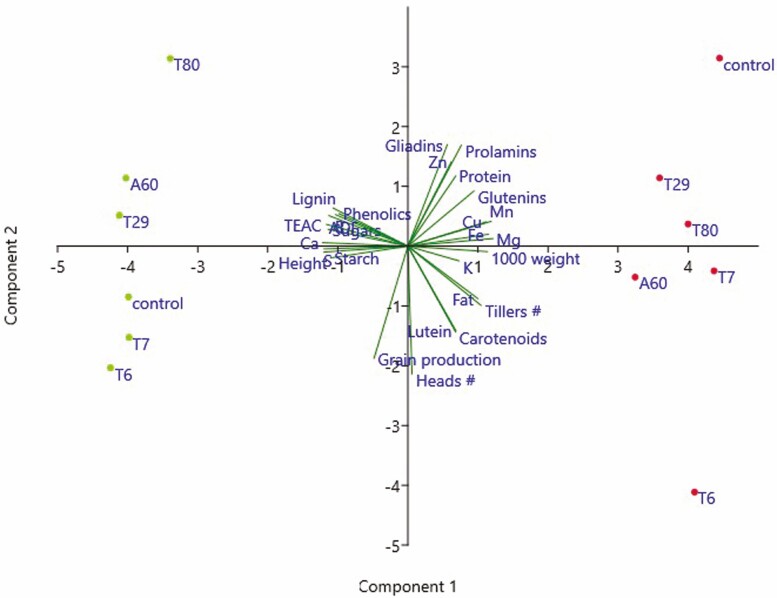
Principal component analysis of growth, yield and grain quality traits of tritordeum cv Aucan plants in Salamanca (red dots) and Córdoba (green dots) field trials including selected inoculation treatments: *Diaporthe* T6, *Pleosporales T7*, Alternaria A60, *Zygomycota T29* and T80, control = uninoculated. Dispersion of samples in the plane defined by components 1 and 2. TEAC = Trolox equivalent antioxidant capacity; DMD = dry matter digestibility; ADF = acid detergent fibre; NDF = neutral detergent fibre. A colour version of this figure appears in the online version of this article.

Results of a PCA analysis including plant growth, grain yield and grain nutritional quality parameters are presented in Fig. 8 (**see**Supporting information Fig. S1). The distribution of samples in the plane defined by PC1 (62.2% of variance) and PC2 (16% of variance) was similar to that obtained only for grain quality parameters (Fig. 7). The main difference was related to PC2, where the number of heads and grain yield were the variables with the highest load in PC2. This changed the distribution of some treatments throughout PC2. However, the position of *Diaporthe* T6 in both field locations remained at the most negative part of PC2 and was associated with the number of heads, grain yield and total lutein and carotenoid content of grain (Fig. 8). These results indicate that the *Diaporthe* T6 treatment increased grain yield and some quality parameters.

## Discussion

The aim of this research was to assess whether symbiotic fungi from *C. gigantea*, a wild grass adapted to a nutrient-poor habitat, could be suitable for improving the field production of tritordeum. Of all 12 fungi assessed, only *Diaporthe* T6 strain significantly increased several growth and grain yield traits of tritordeum under field conditions. In a previous greenhouse bioassay of more than 60 fungal strains isolated from roots of *C. gigantea*, *Diaporthe* T6 was the strain causing the greatest root and shoot growth increment in tritordeum plants (Vázquez de Aldana *et al.* 2021). Thus, the growth promotion observed with *Diaporthe* T6 in a greenhouse bioassay was consistent with a field trial. However, for the remaining strains there was a considerable divergence between the greenhouse and field results. Short-term greenhouse bioassays are important steps to evaluate fungal strain potential for plant growth promotion traits. However, under field conditions biotic and abiotic factors can modify the effects of plant-fungal interactions observed under greenhouse conditions. For instance, inoculants with a positive effect in greenhouse assays like *Paraconiothirium* T33 (Vázquez de Aldana *et al.* 2021) showed a negative effect under field conditions. This inconsistency could be due to the absence of natural soil microbiota in the greenhouse assays, where the substrate used to grow the plants was previously sterilized. In contrast, field soil microbiota is rich, and interactions among its components can affect microbial species abundance and physiology (Markalanda *et al.* 2022). In turn, abiotic environmental parameters can also interact with microbiome components, its structure and plant effects (Bastías *et al.* 2022).

Of all inoculation treatments, *Diaporthe* T6 was the highest-ranked biostimulant within our experiments with tritordeum. The genus *Diaporthe* contains numerous species that can behave as plant pathogens, saprobes or endophytes (Gomes *et al.* 2013; Norphanphoun *et al.* 2022). The *Diaporthe* T6 strain was recently identified as a member of a newly described species, *Diaporthe iberica* (Toghueo *et al.* 2023). *D. iberica* T6 has several *in vitro* characteristics that could be related to plant growth promotion, for instance, this strain can produce ammonium as an end product of protein degradation, has amylase and cellulase activities, which can degrade complex carbohydrates, and siderophores, which can sequester iron (Toghueo *et al.* 2023). Such properties could favour plant growth by increasing the availability of nutrients in the rhizosphere. In addition, *Diaporthe* T6 produces 3-indole acetic acid (IAA), a phytohormone involved in cell elongation and induction of lateral root formation. IAA production by some *Diaporthe* strains has been related to the stimulation of plant growth (Chithra *et al.* 2017; Toghueo *et al.* 2022). Therefore, improved nutrient availability coupled with hormonal growth stimulation could be a mechanism responsible for the plant growth promotion observed in both the field and greenhouse experiments. The absence of fungal structures in roots of tritordeum and tomato inoculated with *Diaporthe* strains suggested that this fungus does not behave as an endophyte in tritordeum (Toghueo *et al.* 2022; Pereira *et al.* 2023). An epiphytic or rhizospheric lifestyle could explain how *Diaporthe* affects plant growth by means of improving nutrient availability and/or hormonal stimulation.

The beneficial effects of *Diaporthe* T6 in the colder location (Salamanca) were related to the number of tillers at the vegetative plant stage, and to the number of heads per plant and grain yield. As we observed, grain yield is usually correlated with the number of heads (Murphy *et al.* 2018; Marcos-Barbero *et al.* 2021). A substantial number of studies reported the beneficial effects of fungal symbionts on the performance of a wide range of plant hosts, but very few of these were based on long-term or field experiments, and even less analysed the effect of fungal symbiosis on grain production or quality. A greenhouse study reported an 11%–50% increase in the grain yield of barley caused by a *Cladosporium* strain (Murphy *et al.* 2018). In potted plant outdoor experiments, *Piriformospora indica* increased barley grain yield by 4%–10% (Achatz *et al.* 2010), and *Trametes versicolor* increased wheat grain yield by 8%–37% (Taghinasab *et al.* 2018). The increments in grain yield reported in the above studies are far from the 60% observed with *Diaporthe* T6 under field conditions in the colder location, and this highlights the agricultural potential of this fungal strain. Interestingly several treatments increased the content of nutrients such as P, S and Cu at both locations. In addition to the role above proposed as nutrient recyclers, fungal symbionts can enhance plant nutrient availability by secreting compounds that solubilize nutrients not available to plants in certain soils (Rai *et al.* 2014; Sarkar *et al.* 2021). Improved plant nutrition can help the plant to cope with environmental stresses. Enhancing nutrient content using inoculation treatments could be a promising management strategy to improve the quality of forage.

Regarding grain nutrient quality, the multivariate analysis showed that inoculation treatments were more effective in the colder location, where the control plants differed more notably from the inoculation treatments, probably due to differences in agroclimatic conditions. At both locations, the *Diaporthe* T6 treatment stood out from the rest, improving grain quality due to an increase in carotenoid content, mainly lutein. In wheat grains, the antioxidant activity of carotenoids protects the seed from deterioration and contributes to a successful germination process (Howitt and Pogson 2006). Dietary carotenoids contribute important biological activities: antioxidant, inhibition of carcinogenesis, enhancement of the immune response, cell defense against reactive oxygen species (ROS) and free radicals, and a reduction in the risk for developing cardiovascular and other degenerative diseases (Briton *et al.* 2009). Lutein is the main carotenoid found in the *Triticum* genus (Hentschel *et al.* 2002), but tritordeum has a carotenoid content 5–8 times higher than durum wheat, and a high proportion of lutein esterified with fatty acids (including both lutein monoesters and diesters) (Atienza *et al.* 2007; Mellado-Ortega and Hornero-Mendez 2012). In tritordeum, a higher content of lutein esters led to an enhanced stability, slower degradation, and subsequently, a greater carotenoid retention throughout grain storage and processing (Mellado-Ortega and Hornero-Mendez 2012, 2017). Strikingly, several inoculation treatments (*Diaporthe* T6, *Drechlera* T7, *Zygomycota T29* and T80) increased free and esterified lutein contents in the colder and drier locations. In the warmer location, the quality of the gluten protein improved with several treatments, particularly the total gliadins and the HMW, which are major determinants of the breadmaking quality. Gliadins and glutenins are the two main components of the gluten fraction of wheat seeds and are responsible for wheat dough properties. Gliadins confer extensibility and viscosity to the dough, while glutenins contribute to its elasticity (Vaquero *et al.* 2018). Agroclimatic conditions may again explain the differential effect of inoculation treatments on the storage protein content of the grain, which increased in the warmer location and decreased in the colder one with respect to the control. In general, a high storage protein content is associated with higher quality.

## Conclusions

The results of this study show the importance of assessing fungal symbionts under field conditions. We assessed 12 symbiotic fungi, with plant growth promotional traits, for their ability to promote the growth of tritordeum under field conditions across two locations in Spain. Only one of these fungal strains, *Diaporthe iberica* strain T6 had a significant effect as a biostimulant in tritordeum and in one location. In terms of grain nutritional quality, inoculation treatments differed between locations. The field vs. greenhouse divergences observed could be largely attributed to the absence of a natural soil microbiota in controlled greenhouse conditions, where inundative inoculation methods are often used in the absence of other microorganisms. Therefore, the use of methods for inoculation tests using soils with their own natural microbiota might be useful to improve the accuracy of short-term bioassays to screen for growth-promoting fungi.

## Supporting Information

The following additional information is available in the online version of this article –

**Table S1**. Fungal strains used for the field experiment, and accession codes of the ITS1-5.8s rDNA-ITS2 nucleotide sequences which were used to approximate a taxonomic identification. For the species identification of strain T6 additional gene sequences were used.

**Table S2**. Soil characteristics of the field-plot locations.

**Table S3**. Nutrient content in leaves of tritordeum plants at the vegetative stage, inoculated with different fungal strains, in Salamanca (SA) and Córdoba (CO) field plots. TEAC: for Trolox equivalent antioxidant capacity (mean ± SE; *n* = 6).

**Table S4.** Chemical composition of grain of tritordeum cv. Aucan, inoculated with strains *Diaporthe* T6; *Pleosporales T7*, A60; *Zygomycota T80*, T29, in Salamanca and Córdoba field plots (mean ± SE). The variation of each treatment respect to the uninoculated control is marked in blue > 8%, or in red < 8%.

**Table S5.** Fatty acids of grain of tritordeum cv. Aucan, inoculated with fungal strains *Diaporthe* T6; *Pleosporales T7*, A60; *Zygomycota T80*, T29, in Salamanca and Córdoba field plots (mean ± SE; *n* = 3)

**Figure S1.** Pearson´s correlation coefficients (*r*) among plant growth, grain yield and grain quality parameters across both locations and selected inoculation treatments included in the PCA. The *r* values are indicated by the size of the circle and the colour according to the scale. Only significant correlations with a *P* value < 0.05 are included.

plae013_suppl_Supplementary_Materials

## Data Availability

The data underlying this article are available in DIGITAL.CSIC, at https://dx.doi.org/10.20350/digitalCSIC/15720
