# Supplementary material for: Symbiotic fungi from a wild grass (Celtica gigantea) increase the growth, grain yield and quality of tritordeum under field conditions
Source: AoB Plants. 2024 Mar 15;16(2):plae013. doi: 10.1093/aobpla/plae013 (PMC11005784; doi:10.1093/aobpla/plae013)

SUPPLEMENTARY MATERIAL

Table S1. Fungal strains used for the field experiment, and accession codes of the ITS1-5.8s rDNA-ITS2 nucleotide sequences which were used to approximate a taxonomic identification. For the species identification of strain T6 additional gene sequences were used.

Strain ID	Taxon	GenBank accession number	Reference
T6	*Diaporthe iberica*	MT645115	Toghueo et al. 2023<br>Vázquez de Aldana et al. 2021
T61	*Diaporthe*	MT645115	Vázquez de Aldana et al. 2021
FR9	*Diaporthe*	n.a.	
A60	*Alternaria*	MT645101	Vázquez de Aldana et al. 2021
T7	*Pleosporales*	MT645101	Vázquez de Aldana et al. 2021
T17	*Collembolispora*	MT645107	Vázquez de Aldana et al. 2021
T33	*Paraconiothirium*	MT645131	Vázquez de Aldana et al. 2021
T10	*Dothideomycota*	MT645137	Vázquez de Aldana et al. 2021
T40	*Basidiomycota*	MT645125	Vázquez de Aldana et al. 2021
T29	*Zygomycota*	MT645148	Vázquez de Aldana et al. 2021
T80	*Zygomycota*	MT645148	Vázquez de Aldana et al. 2021
T18	*unknown*	n.a.	

n.a.: not available

Table S2. Soil characteristics of the field-plot locations.

	**Salamanca**	**Córdoba**
Coordinates	40°54’16’’ N<br>5°46’32’’ W	37°51’54’’ N<br>4°47’53’’ W
Altitude (m)	798	106
pH	7.31	7.79
Organic matter (%)	1.75	7.57
N (%)	0.078	0.043
C (%)	1.02	4.39
C:N	13.0	102
P (ppm)	36.7	34.7
Ca (ppm)	2750	14773
K (ppm)	164	169
Mg (ppm)	354	274

Table S3. Nutrient content in leaves of tritordeum plants at the vegetative stage, inoculated with different fungal strains, in Salamanca (SA) and Córdoba (CO) field plots. TEAC: for Trolox equivalent antioxidant capacity (mean ± SE; n=6).

**Fungal strain**	**Plot**	**N (g/kg)**	**P (g/kg)**	**K (g/kg)**	**Ca (g/kg)**	**Mg (g/kg)**	**S (g/kg)**	**Fe (mg/kg)**	**Mn (mg/kg)**	**Cu (mg/kg)**	**Zn (mg/kg)**	**TEAC (µEq Tr/g)**
Control	SA	20.21 ±1.81	1.88 ±0.01	8.55 ±0.04	2.37 ±0.55	0.83 ±0.04	0.72 ±0.14	43.05 ±5.85	11.25 ±0.05	3.28 ±0.49	6.64 ±0.32	10.05 ±2.29
Diaporthe T6	SA	19.62 ±0.83	1.74 ±0.07	7.56 ±0.90	1.84 ±0.08	0.80 ±0.09	0.58 ±0.11	37.25 ±4.95	11.55 ±0.55	2.32 ±0.20	6.03 ±0.10	10.13 ±0.15
Diaporthe T61	SA	18.23 ±2.42	1.87 ±0.30	8.02 ±0.49	1.84 ±0.05	0.72 ±0.04	0.72 ±0.16	36.45 ±0.65	10.75 ±0.65	3.00 ±1.10	6.82 ±0.54	11.13 ±0.28
Diaporthe FR9	SA	20.05 ±1.01	2.49 ±0.04	8.85 ±0.06	2.00 ±0.01	0.85 ±0.05	1.24 ±0.34	62.20 ±9.70	12.90 ±0.80	4.41 ±0.83	7.88 ±0.55	11.31 ±1.11
Alternaria A60	SA	20.22 ±0.31	1.94 ±0.09	8.50 ±0.26	1.98 ±0.24	0.72 ±0.01	0.54 ±0.03	37.15 ±1.05	13.15 ±0.65	2.82 ±0.49	6.56 ±0.58	10.01 ±0.89
Pleosporales T7	SA	19.41 ±1.21	1.95 ±0.12	8.73 ±0.39	2.13 ±0.41	0.86 ±0.05	0.91 ±0.16	40.50 ±1.70	11.65 ±0.65	3.40 ±0.06	7.34 ±0.52	10.65 ±0.64
Collembolispora T17	SA	21.44 ±4.01	2.12 ±0.23	8.48 ±0.29	2.08 ±0.26	0.75 ±0.01	0.81 ±0.14	41.60 ±4.20	11.40 ±1.70	3.37 ±0.24	7.25 ±0.65	10.29 ±0.88
Microsphaeropsis T33	SA	17.87 ±1.72	2.32 ±0.26	8.13 ±0.93	1.85 ±0.11	0.74 ±0.09	0.71 ±0.16	49.45 ±4.65	10.70 ±0.60	10.44 ±6.25	8.54 ±2.00	8.78 ±0.41
Dothideomycota T10	SA	21.23 ±4.21	2.32 ±0.71	10.00 ±2.39	3.00 ±0.57	1.00 ±0.16	1.26 ±0.09	50.90 ±10.50	16.30 ±5.00	3.57 ±0.28	8.03 ±1.41	9.58 ±0.71
Basidiomycota T40	SA	19.58 ±1.21	2.46 ±0.11	8.76 ±0.52	1.98 ±0.16	0.74 ±0.01	1.07 ±0.15	44.05 ±1.25	12.20 ±2.00	4.66 ±1.80	6.54 ±0.63	9.85 ±0.53
Zygomycota T80	SA	19.21 ±2.32	2.04 ±0.17	7.99 ±0.03	1.81 ±0.17	0.70 ±0.02	0.86 ±0.07	41.20 ±7.50	11.35 ±0.95	3.87 ±0.54	6.67 ±0.98	10.69 ±1.63
Zygomycota T29	SA	21.83 ±2.63	2.54 ±0.72	10.03 ±0.57	2.68 ±0.36	0.89 ±0.02	1.21 ±0.43	45.10 ±4.70	15.85 ±3.25	3.82 ±0.16	7.61 ±0.19	9.87 ±0.36
Unknown T18	SA	16.09 ±2.12	2.23 ±0.87	8.59 ±2.10	1.29 ±0.58	0.60 ±0.02	1.04 ±0.28	41.90 ±4.30	11.60 ±2.31	3.80 ±1.20	6.75 ±1.90	11.39 ±0.98
Mean	SA	19.83 ±0.37	2.16 ±0.08	8.88 ±0.21	2.24 ±0.09	0.78 ±0.02	0.96 ±0.06	49.32 ±1.79	12.86 ±0.49	4.11 ±0.52	6.38 ±0.21	10.42 ±0.50
Control	CO	28.32 ±3.12	1.27 ±0.19	14.10 ±1.65	2.74 ±0.31	0.57 ±0.07	2.07 ±0.44	70.78 ±8.56	15.90 ±2.30	7.89 ±0.78	21.78 ±2.70	9.24 ±0.38
Diaporthe T6	CO	26.83 ±2.71	1.71 ±0.18	16.92 ±1.45	3.56 ±0.29	0.81 ±0.09	3.57 ±1.20	92.93 ±13.19	22.85 ±1.67	16.33 ±4.92	31.73 ±3.20	9.51 ±0.55
Diaporthe T61	CO	25.67 ±1.81	1.38 ±0.18	14.34 ±1.55	3.03 ±0.29	0.65 ±0.06	2.06 ±0.36	74.35 ±5.68	17.73 ±1.93	15.67 ±5.52	22.97 ±1.78	9.28 ±0.38
Diaporthe FR9	CO	28.54 ±2.52	1.53 ±0.20	16.02 ±1.57	3.23 ±0.41	0.72 ±0.10	2.20 ±0.33	74.40 ±4.19	20.25 ±2.76	7.89 ±0.55	25.58 ±3.25	9.01 ±0.44
Alternaria A60	CO	25.97 ±2.02	1.42 ±0.19	14.56 ±1.76	2.58 ±0.26	0.57 ±0.06	1.95 ±0.28	72.60 ±6.51	17.68 ±1.85	9.17 ±9.17	19.83 ±2.01	9.34 ±0.44
Pleosporales T7	CO	24.52 ±1.54	1.47 ±0.18	14.16 ±1.49	2.84 ±0.29	0.60 ±0.05	1.91 ±0.33	73.52 ±4.33	17.27 ±2.33	11.44 ±3.17	22.12 ±1.80	9.63 ±0.44
Collembolispora T17	CO	23.94 ±2.05	1.51 ±0.11	16.04 ±0.81	3.28 ±0.27	0.67 ±0.04	2.41 ±0.27	70.48 ±3.15	18.76 ±1.49	10.32 ±1.25	28.62 ±2.96	9.44 ±0.36
Microsphaeropsis T33	CO	26.91 ±2.51	1.34 ±0.14	14.32 ±1.35	2.93 ±0.29	0.65 ±0.09	1.99 ±0.38	74.78 ±7.00	17.73 ±2.12	11.65 ±2.43	27.42 ±3.34	9.20 ±0.49
Dothideomycota T10	CO	26.82 ±3.02	1.61 ±0.25	15.00 ±1.83	2.75 ±0.28	0.62 ±0.07	2.09 ±0.42	69.72 ±6.84	18.44 ±2.35	14.27 ±3.69	23.84 ±2.17	9.86 ±0.64
Basidiomycota T40	CO	26.97 ±1.91	1.54 ±0.24	14.71 ±1.47	3.22 ±0.39	0.64 ±0.08	2.16 ±0.37	75.73 ±7.53	17.48 ±1.78	13.85 ±6.03	27.00 ±4.23	9.40 ±0.24
Zygomycota T80	CO	27.71 ±1.91	1.46 ±0.17	16.23 ±2.18	3.43 ±0.41	0.80 ±0.12	2.35 ±0.49	82.78 ±13.06	20.77 ±2.53	13.25 ±2.38	29.15 ±6.58	9.60 ±0.52
Zygomycota T29	CO	26.72 ±1.22	1.25 ±0.14	12.95 ±1.04	2.72 ±0.15	0.62 ±0.05	1.91 ±0.27	68.98 ±5.61	17.03 ±1.02	11.83 ±0.99	22.85 ±1.35	9.03 ±0.53
Unknown T18	CO	29.03 ±3.83	1.38 ±0.30	14.09 ±2.43	2.68 ±0.49	0.58 ±0.12	2.03 ±0.49	65.35 ±6.53	16.15 ±3.45	8.05 ±1.65	18.65 ±2.65	9.02 ±1.21
Mean	CO	26.53 ±0.37	1.47 ±0.05	15.00 ±0.42	3.03 ±0.09	0.66 ±0.02	2.24 ±0.09	75.15 ±1.82	18.56 ±0.47	11.99 ±0.78	25.04 ±0.88	9.39 ±0.31

Table S4. Chemical composition of grain of tritordeum cv. Aucan, inoculated with strains *Diaporthe* T6; *Pleosporales T7*, A60; *Zygomycota T80*, T29, in Salamanca and Córdoba field plots (mean ±SE). The variation of each treatment respect to the uninoculated control is marked in blue >8%, or in red <8%.

	**Salamanca**						**Córdoba**					
	**Control**	**T6**	**T7**	**A60**	**T80**	**T29**	**Control**	**T6**	**T7**	**A60**	**T80**	**T29**
Fat (g/100g) (n=3)	2.39 ±0.01	2.45 ±0.10	2.36 ±0.02	2.31 ±0.09	2.35 ±0.07	2.26 ±0.03	2.07 ±0.06	2.24 ±0.02	2.28 ±0.05	2.05 ±0.04	2.08 ±0.09	2.14 ±0.04
Starch (g/100g)(n=3)	34.5 ±0.75	34.7 ±0.70	32.8 ±1.20	37.0 ±0.01	34.6 ±1.00	34.4 ±0.90	38.5 ±1.00	38.6 ±0.25	39.2 ±1.30	38.9 ±1.05	37.9 ±1.10	37.0 ±0.20
Protein (g/100g) (n=6)	14.8 ±0.49	14.5 ±0.09	13.9 ±0.09	14.0 ±0.75	14.1 ±0.08	14.2 ±0.06	13.2 ±0.50	12.7 ±0.60	13.1 ±0.64	13.4 ±0.56	14.7 ±0.75	14.3 ±0.65
Soluble sugars (n=3)												
Fructose (g/100g)	0.45 ±0.05	0.55 ±0.05	0.45 ±0.05	0.55 ±0.05	0.45 ±0.05	0.65 ±0.15	0.65 ±0.05	0.65 ±0.15	0.70 ±0.10	0.60 ±0.10	0.70 ±0.01	0.75 ±0.05
Glucose (g/100g)	0.50 ±0.00	0.65 ±0.05	0.50 ±0.00	0.50 ±0.00	0.50 ±0.00	0.50 ±0.00	0.75 ±0.05	0.85 ±0.15	0.75 ±0.05	0.75 ±0.05	0.80 ±0.10	1.05 ±0.05
Sacarose (g/100g)	0.40 ±0.00	0.40 ±0.00	0.40 ±0.00	0.40 ±0.00	0.40 ±0.00	0.40 ±0.00	0.40 ±0.00	0.40 ±0.00	0.40 ±0.00	0.40 ±0.00	0.40 ±0.00	0.40 ±0.00
Maltose (g/100g)	1.40 ±0.10	1.30 ±0.10	1.45 ±0.05	1.40 ±0.00	1.35 ±0.05	1.35 ±0.05	1.65 ±0.05	1.50 ±0.10	1.55 ±0.05	1.85 ±0.05	1.80 ±0.10	1.75 ±0.05
Lactose (g/100g)	0.40 ±0.00	0.40 ±0.00	0.40 ±0.00	0.40 ±0.00	0.40 ±0.00	0.40 ±0.00	0.40 ±0.00	0.40 ±0.00	0.40 ±0.00	0.40 ±0.00	0.40 ±0.00	0.40 ±0.00
Total sugars (g/100g)	2.35 ±0.05	2.50 ±0.20	2.40 ±0.00	2.45 ±0.15	2.30 ±0.10	2.50 ±0.10	3.05 ±0.05	3.00 ±0.40	3.00 ±0.20	3.20 ±0.20	3.30 ±0.20	3.55 ±0.05
Active gliadins (n=3)												
Omega (µg/mg)	4.69 ±0.49	3.54 ±0.20	4.98 ±0.40	4.43 ±0.66	4.54 ±0.28	5.00 ±0.34	3.23 ±0.18	4.19 ±0.62	4.08 ±0.48	3.90 ±0.63	5.36 ±0.54	5.25 ±0.24
Alfa (µg/mg)	30.9 ±2.11	25.8 ±0.99	31.7 ±1.19	30.6 ±3.05	31.4 ±1.27	31.2 ±1.66	24.3 ±0.38	26.1 ±1.16	27.2 ±0.84	26.9 ±1.74	31.2 ±1.70	29.7 ±0.76
Gamma (µg/mg)	20.4 ±2.29	15.8 ±0.74	21.2 ±0.57	18.3 ±2.04	20.7 ±0.78	20.7 ±0.87	16.9 ±0.27	17.4 ±0.62	17.9 ±0.38	18.3 ±0.88	20.1 ±0.98	19.3 ±0.56
Gliadins (µg/mg)	56.1 ±4.86	45.1 ±1.60	57.8 ±2.16	53.3 ±5.66	56.7 ±2.26	56.9 ±2.85	44.5 ±0.83	47.7 ±2.40	49.2 ±1.71	49.1 ±3.20	56.6 ±3.22	54.2 ±1.56
Glutenins (n=3)												
HMW (µg/mg)	5.07 ±0.07	4.79 ±0.16	4.95 ±0.22	4.61 ±0.11	5.11 ±0.28	4.94 ±0.13	4.17 ±0.03	4.46 ±0.21	4.53 ±0.06	5.53 ±0.30	5.39 ±0.34	4.58 ±0.08
LMW (µg/mg)	18.0 ±0.41	17.1 ±0.28	16.2 ±0.22	16.4 ±0.52	16.1 ±0.29	15.8 ±1.35	15.0 ±0.21	12.3 ±0.07	13.0 ±0.49	14.8 ±0.76	15.7 ±0.77	13.7 ±0.30
Glutenins (µg/mg)	23.1 ±0.48	21.9 ±0.13	21.1 ±0.45	20.9 ±0.64	21.2 ±0.23	20.7 ±1.46	19.2 ±0.23	16.7 ±0.23	17.5 ±0.48	20.3 ±1.05	21.1 ±1.11	18.3 ±0.34
Prolamins (µg/mg)	79.2 ±4.81	67.1 ±1.58	78.9 ±2.50	74.3 ±5.07	77.9 ±2.47	77.6 ±2.72	63.7 ±1.03	64.5 ±2.41	66.7 ±1.31	69.5 ±4.26	77.7 ±4.32	72.5 ±1.44
Pigments												
Trans-lutein (µg/g)	1.48	2.19	1.86	1.37	1.72	1.72	1.43	1.42	1.46	1.42	1.39	1.57
Cis-lutein(µg/g)	0.36	0.44	0.38	0.30	0.36	0.34	0.32	0.31	0.33	0.34	0.32	0.29
Lutein monosteres(µg/g)	0.24	0.32	0.58	0.47	0.53	0.53	0.61	0.58	0.36	0.33	0.33	0.33
Lutein disteres (µg/g)	0.01	0.01	0.08	0.07	0.07	0.07	0.10	0.09	0.04	0.03	0.03	0.03
Trans-βcarotene (µg/g)	0.02	0.02	0.02	0.01	0.02	0.01	0.01	0.01	0.01	0.02	0.01	0.02
Total lutein (µg/g)	2.09	2.96	2.89	2.21	2.68	2.66	2.46	2.40	2.19	2.12	2.07	2.22
Total carotenoids(µg/g)	2.10	2.98	2.91	2.22	2.69	2.67	2.47	2.41	2.22	2.13	2.08	2.24
Total phenolics (mg/g) (n=6)	0.94 ±0.02	0.94 ±0.04	0.90 ±0.06	0.91 ±0.04	0.92 ±0.05	0.95 ±0.02	0.98 ±0.03	0.98 ±0.04	0.97 ±0.05	0.98 ±0.04	1.02 ±0.04	1.06 ±0.03
TEAC (µEqTrolox/g) (n=6)	1.32 ±0.06	1.29 ±0.10	1.27 ±0.02	1.31 ±0.04	1.21 ±0.05	1.36 ±0.06	1.54 ±0.04	1.52 ±0.02	1.50 ±0.12	1.54 ±0.04	1.58 ±0.05	1.50 ±0.05

	Salamanca						Córdoba					
	Control	T6	T7	A60	T80	T29	Control	T6	T7	A60	T80	T29
Minerals (n=6)												
P (g/kg)	3.22 ±0.12	3.22 ±0.05	3.17 ±0.12	3.04 ±0.08	3.28 ±0.12	3.31 ±0.09	3.54 ±0.14	3.38 ±0.09	3.51 ±0.07	3.67 ±0.11	3.57 ±0.13	3.44 ±0.08
K (g/kg)	3.27 ±0.11	3.39 ±0.13	3.34 ±0.11	3.18 ±0.13	3.25 ±0.09	3.49 ±0.10	3.37 ±0.10	3.07 ±0.08	3.15 ±0.07	3.15 ±0.07	3.15 ±0.10	3.18 ±0.07
Ca (g/kg)	0.32 ±0.01	0.31 ±0.01	0.31 ±0.01	0.29 ±0.01	0.31 ±0.01	0.33 ±0.01	0.77 ±0.02	0.76 ±0.05	0.71 ±0.03	0.77 ±0.03	0.70 ±0.03	0.75 ±0.02
Mg (g/kg)	1.05 ±0.03	1.00 ±.05	1.01 ±0.07	0.96 ±0.04	1.00 ±0.06	1.02 ±0.05	0.71 ±0.05	0.69 ±0.04	0.72 ±0.02	0.75 ±0.03	0.72 ±0.03	0.70 ±0.03
S (g/kg)	0.19 ±0.01	0.24 ±0.02	0.21 ±0.01	0.18 ±0.02	0.22 ±0.01	0.23 ±0.01	0.93 ±0.12	0.73 ±0.11	0.78 ±0.09	0.70 ±0.06	0.75 ±0.09	0.81 ±0.08
Fe (mg/kg)	33.8 ±1.10	33.4 ±1.71	32.5 ±2.34	30.1 ±1.16	33.7 2.01	38.3 ±4.6	23.9 ±1.17	23.6 ±1.05	23.8 ±0.88	24.1 ±1.56	24.7 ±1.10	25.2 ±0.60
Mn (mg/kg)	25.2 ±0.83	23.9 ±1.18	23.8 ±2.01	22.7 ±1.52	24.8 ±1.51	24.4 ±0.90	18.7 ±1.03	17.4 ±1.01	18.2 ±0.80	18.3 ±0.78	20.1 ±0.64	19.4 ±0.76
Cu (mg/kg)	9.30 ±1.13	7.53 ±1.09	6.94 ±1.31	9.34 ±1.85	7.49 ±1.22	9.02 ±1.41	4.72 ±0.38	4.45 0.24	4.94 ±0.41	4.49 ±0.15	5.43 ±0.62	4.43 ±0.18
Zn (mg/kg)	31.9 ±2.19	26.8 ±1.07	27.4 ±1.87	27.1 ±1.33	29.6 ±1.95	29.3 ±2.21	27.4 ±2.07	26.7 ±2.27	27.9 ±1.62	27.2 ±1.12	27.4 ±1.84	27.1 ±2.59
Fibers (n=6)												
ADF (%)	3.19 ±0.12	3.08 ±0.12	3.22 ±0.08	3.18 ±0.08	3.31 ±0.07	3.45 ±0.10	3.73 ±0.17	3.55 ±0.14	3.71 ±0.14	3.66 ±0.12	3.69 ±0.11	3.78 ±0.14
NDF (%)	15.6 ±1.23	15.1 ±0.61	14.4 ±0.32	15.9 ±0.97	16.5 ±1.23	14.4 ±0.41	15.7 ±0.87	14.6 ±0.21	14.5 ±0.34	14.7 ±0.35	14.8 ±0.30	15.9 ±0.86
Lignin (%)	0.93 ±0.10	0.86 ±0.07	0.98 ±0.07	0.85 ±0.04	0.98 ±0.08	1.09 ±0.09	1.12 ±0.07	1.16 ±0.11	1.18 ±0.12	1.18 ±0.12	1.21 ±0.12	1.24 ±0.07
DMD (%)	74.9 ±0.75	75.4 ±0.55	75.4 ±0.33	75.0 ±0.45	74.3 ±0.43	75.2 ±0.65	74.7 ±0.43	74.9 ±0.37	75.2 ±0.47	74.9 ±0.70	74.7 ±0.64	73.9 ±0.58

TEAC= Trolox equivalent antioxidant capacity; DMD= dry matter digestibility; ADF= acid detergent fiber; NDF= neutral detergent fiber

Table S5. Fatty acids of grain of tritordeum cv. Aucan, inoculated with fungal strains *Diaporthe* T6; *Pleosporales T7*, A60; *Zygomycota T80*, T29, in Salamanca and Córdoba field plots (mean ±SE; n=3)

	**Salamanca**						**Córdoba**					
	**Control**	**T6**	**T7**	**A60**	**T80**	**T29**	**Control**	**T6**	**T7**	**A60**	**T80**	**T29**
Fatty acids (g/100g)												
Lauric	0.10 ±0.00	0.10 0.00±	0.10 ±0.00	0.10 ±0.00	0.10 ±0.00	0.10 ±0.00	0.10 ±0.00	0.10 ±0.00	0.10 ±0.00	0.10 ±0.00	0.10 ±0.00	0.10 ±0.00
Mystiric	0.10 ±0.00	0.10 ±0.00	0.10 ±0.00	0.15 ±0.05	0.10 ±0.00	0.10 ±0.00	0.10 ±0.00	0.10 ±0.00	0.10 ±0.00	0.10 ±0.00	0.10 ±0.00	0.10 ±0.00
Palmitic	16.4 ±0.15	16.0 ±0.05	16.3 ±0.10	18.2 ±1.90	16.2 ±0.05	16.2 ±0.00	16.5 ±0.15	16.5 ±0.05	16.6 ±0.00	16.6 ±0.05	16.9 ±0.10	16.8 ±0.10
Palmitoleic	0.30 ±0.00	0.30 ±0.00	0.30 ±0.00	0.35 ±0.05	0.35 ±0.05	0.30 ±0.00	0.25 ±0.05	0.30 ±0.00	0.30 ±0.00	0.25 ±0.05	0.25 ±0.05	0.30 ±0.00
Margaric	0.10 ±0.00	0.10 ±0.00	0.10 ±0.00	0.10 ±0.00	0.10 ±0.00	0.10 ±0.00	0.10 ±0.00	0.10 ±0.00	0.10 ±0.00	0.10 ±0.00	0.10 ±0.00	0.10 ±0.00
Margaroleic	0.25 ±0.05	0.40 ±0.10	0.25 ±0.05	0.15 ±0.05	0.25 ±0.10	0.25 ±0.10	0.35 ±0.15	0.30 ±0.10	0.30 ±0.00	0.30 ±0.10	0.30 ±0.00	0.35 ±0.15
Stearic	1.70 ±0.00	1.70 ±0.00	1.75 ±0.05	1.90 ±0.20	1.75 ±0.05	1.70 ±0.00	1.30 ±0.00	1.30 ±0.00	1.30 ±0.00	1.30 ±0.00	1.30 ±0.00	1.30 ±0.00
Oleic	22.1 ±0.10	21.6 ±0.05	21.8 ±0.05	23.3 ±1.55	22.2 ±0.05	21.6 ±0.05	18.6 ±0.15	18.4 ±0.05	18.1 ±0.05	18.8 ±0.25	18.3 ±0.10	18.6 ±0.30
Trans-Oleic	0.10 ±0.00	0.10 ±0.00	0.10 ±0.00	0.25 ±0.15	0.10 ±0.00	0.10 ±0.00	0.20 ±0.10	0.10 ±0.00	0.10 ±0.00	0.15 ±0.05	0.10 ±0.00	0.10 ±0.00
Linoleic	54.2 ±0.30	54.6 ±0.10	54.5 0.10	51.0 ±3.65	54.2 ±0.10	54.9 ±0.20	56.7 ±0.80	57.3 ±0.30	57.8 ±0.10	56.6 ±0.65	57.0 ±0.45	57.0 ±0.50
Trans-Linoleic	0.10 ±0.00	0.10 ±0.00	0.10 ±0.00	0.45 ±0.25	0.10 ±0.00	0.10 ±0.00	0.60 ±0.10	0.10 ±0.00	0.10 ±0.00	0.45 ±0.20	0.40 ±0.10	0.10 ±0.00
Linolenic	3.25 ±0.05	3.60 ±0.10	3.40 ±0.10	2.70 ±0.70	3.40 ±0.10	3.45 ±0.15	3.95 ±0.05	4.15 ±0.05	4.00 ±0.00	3.85 ±0.05	3.80 ±0.10	4.00 ±0.10
Trans-linolenic	0.10 ±0.00	0.10 ±0.00	0.10 ±0.00	0.10 ±0.00	0.10 ±0.00	0.10 ±0.00	0.10 ±0.00	0.10 ±0.00	0.10 ±0.00	0.10 ±0.00	0.10 ±0.00	0.10 ±0.00
Araquic	0.30 ±0.00	0.30 ±0.00	0.30 ±0.00	0.30 ±0.00	0.30 ±0.00	0.30 ±0.00	0.20 ±0.00	0.25 ±0.05	0.20 ±0.00	0.25 ±0.05	0.20 ±0.00	0.20 ±0.00
Gadolenic	0.80 ±0.00	0.80 ±0.00	0.80 ±0.00	0.90 ±0.10	0.80 ±0.00	0.80 ±0.00	0.95 ±0.05	0.90 ±0.00	0.90 ±0.00	0.95 ±0.05	0.95 ±0.05	0.95 ±0.05
Behenic	0.20 ±0.00	0.20 ±0.00	0.20 ±0.00	0.20 ±0.00	0.20 ±0.00	0.20 ±0.00	0.15 ±0.05	0.10 ±0.00	0.10 ±0.00	0.15 ±0.05	0.15 ±0.05	0.15 ±0.05
Lignoceric	0.20 ±0.00	0.20 ±0.00	0.20 ±0.00	0.20 ±0.00	0.20 ±0.00	0.20 ±0.00	0.10 ±0.00	0.15 ±0.05	0.10 ±0.00	0.10 ±0.00	0.10 ±0.00	0.15 ±0.05

Figure S1. Pearson´s correlation coefficients (r) between plant growth, grain yield and grain quality parameters across both locations and selected inoculation treatments included in the PCA. The r values are indicated by the size of the circle and the colour according to the scale. Only significant correlations with a p value <0.05 are included.

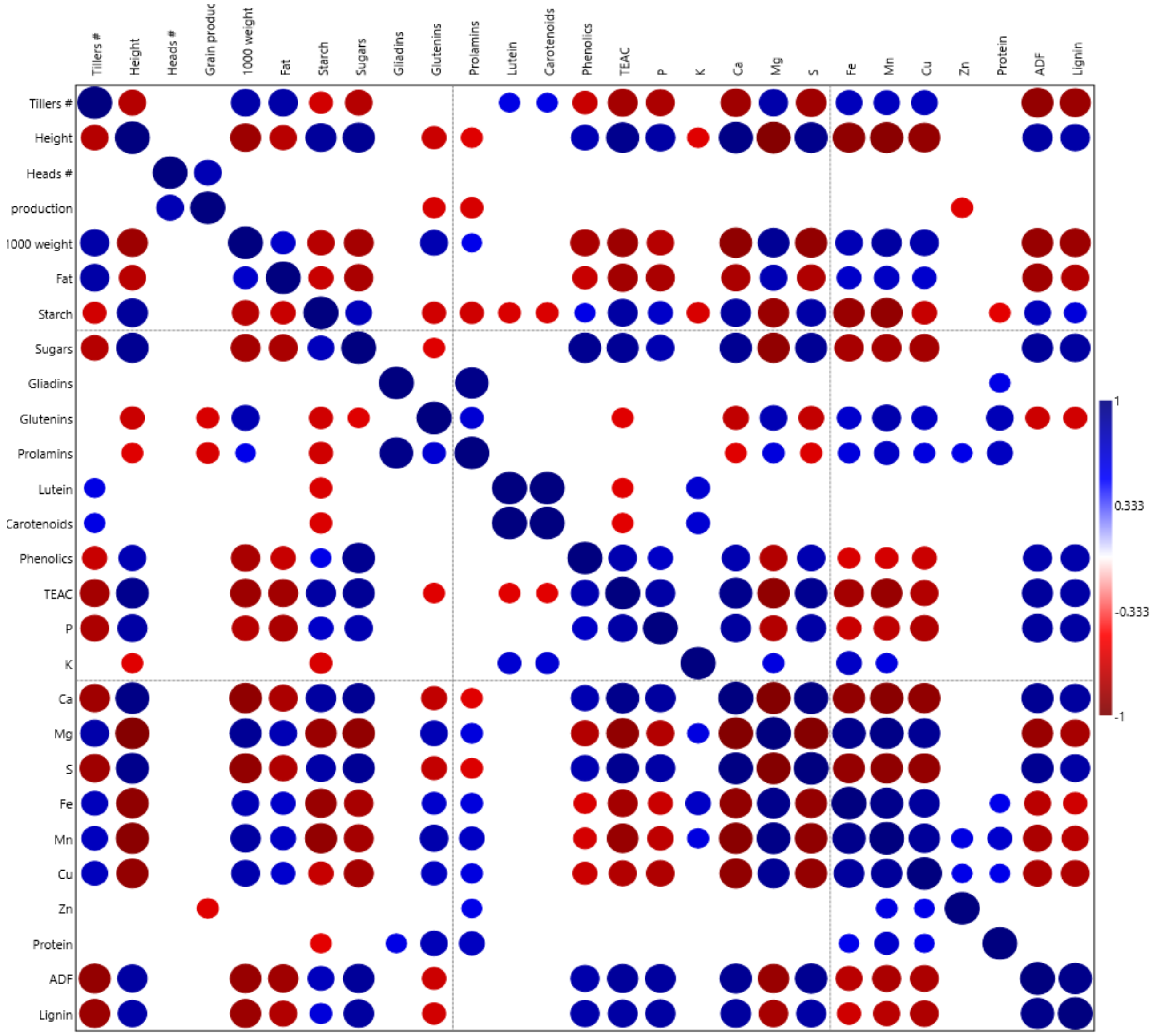

Supplement: plae013_suppl_Supplementary_Materials [file plae013_suppl_supplementary_materials.pdf]
